# Physical Activity and Psychological Well-Being Among Older Adults: A Dual Social–Affective Pathway Model

**DOI:** 10.3390/bs16081381

**Published:** 2026-08-11

**Authors:** Sejoong Lee, Byoungwook Ahn, Chaeman Na

**Affiliations:** 1 Department of Leisure Marine Sports, Seosan Campus, Hanseo University, Seosan-si 31692, Republic of Korea; 2 Department of Physical Education, Metropol Campus, Kyungdong University, Yangju-si 11458, Republic of Korea

**Keywords:** physical activity, psychological well-being, social contact, positive affect, older adults, social–affective pathways

## Abstract

Physical activity is associated with psychological well-being in later life, yet the interpersonal and affective pathways underlying this relationship remain unclear. Using data from the 2020 Korean National Survey of Older Persons, this study examined associations among physical activity, social contact, positive affect, and evaluative psychological well-being. Confirmatory factor analysis and structural equation modeling were conducted using AMOS 26.0, with indirect effects evaluated through bias-corrected bootstrapping with 5000 resamples. Physical activity was positively associated with both social contact and positive affect, which were, in turn, positively associated with psychological well-being. The direct association between physical activity and psychological well-being was not statistically significant (β = 0.019, *p* = 0.077), whereas the total indirect association through social contact and positive affect was significant (β = 0.109, 95% CI [0.094, 0.125], *p* < 0.001). The model explained 29.7% of the variance in psychological well-being. These findings are consistent with a dual interpersonal–affective perspective on the association between physical activity and psychological well-being in later life. Given the cross-sectional design, these associations should be interpreted as statistical rather than causal. The social-contact pathway should be interpreted with caution, as this construct showed limited reliability and convergent validity.

## 1. Introduction

Population aging has become one of the most significant demographic transformations worldwide, creating growing concern regarding how to maintain and enhance psychological well-being in later life. As life expectancy continues to increase, the challenge is no longer limited to extending years of life but also ensuring that older adults experience meaningful, satisfying, and psychologically healthy lives. Consequently, identifying factors that promote psychological well-being has become a major priority for researchers and policymakers in aging societies.

Among the various determinants of successful aging, physical activity has consistently been recognized as one of the most important behavioral resources, associated with higher life satisfaction and lower depression among older adults (McAuley et al., 2000; Netz et al., 2005; Rejeski & Mihalko, 2001); the theoretical basis for this association is discussed in detail in Section 2.1.

However, the association between physical activity and psychological well-being may involve interpersonal and affective processes in addition to individual-level behavioral factors. Participation in physical activity frequently occurs in social contexts where individuals interact with family members, friends, neighbors, or community groups. Such interactions may facilitate the development and maintenance of social relationships, which are themselves important determinants of well-being in later life (Berkman & Glass, 2000; Holt-Lunstad et al., 2010). Accordingly, recent research has increasingly emphasized the socially embedded nature of physical activity and suggested that its benefits may partially operate through social pathways rather than solely through individual-level behavioral processes.

Understanding these pathways requires conceptual precision. Social contact represents an interpersonal or structural dimension of social life, reflecting the extent to which individuals remain connected with others. Positive affect, by contrast, represents an affective state characterized by pleasant emotional experiences such as joy, vitality, and feeling glad to be alive. Although social interaction may contribute to positive affect, the two constructs are not interchangeable: social contact concerns relational connectedness, whereas positive affect concerns emotional experience. Distinguishing these pathways makes it possible to examine whether physical activity is associated with psychological well-being through interpersonal engagement, affective experience, or both.

Physical activity may be associated with positive affect through physiological responses to exercise, enjoyment of valued activities, experiences of competence, and the social context in which activity occurs. The broaden-and-build theory proposes that positive emotions broaden individuals’ momentary thought–action repertoires and help build enduring psychological and social resources (Fredrickson, 2001). Accordingly, positive affect may constitute an affective pathway linking physical activity with evaluative well-being, without being treated as a measure of emotional support or relationship quality.

Socioemotional Selectivity Theory further suggests that emotionally meaningful experiences become increasingly important in later life as perceived future time horizons become more limited (Carstensen et al., 1999). This perspective is relevant to the present model because physical activity may provide both opportunities for interpersonal contact and occasions for positive emotional experience. These dimensions may operate simultaneously while remaining conceptually distinct.

These issues are particularly relevant in the Korean context. South Korea is currently experiencing one of the fastest rates of population aging in the world. Simultaneously, traditional family structures that historically served as the primary source of social support for older adults have undergone substantial transformation. Declining fertility rates, increasing numbers of single-person households, urbanization, and changing intergenerational relationships have weakened traditional support systems and altered patterns of social interaction among older adults. As a result, concerns regarding social isolation, loneliness, and reduced social participation have become increasingly prominent social issues. Under these conditions, understanding how different forms of social relationships contribute to psychological well-being is of both theoretical and practical importance.

Despite growing interest in the relationships among physical activity, social connectedness, affect, and psychological well-being, several gaps remain. First, previous studies have often emphasized direct associations between physical activity and well-being while paying less attention to the mechanisms through which these associations may operate. Second, interpersonal and affective processes are frequently discussed together even though social contact and positive affect represent different constructs. Third, relatively few large-scale studies have tested these two pathways simultaneously among older adults. Addressing these gaps requires a model that separates social connectedness from positive emotional experience.

To address these limitations, the present study proposes a dual social–affective pathway model that distinguishes social contact as an interpersonal dimension from positive affect as an affective dimension. Drawing on Activity Theory, Socioemotional Selectivity Theory, and the broaden-and-build theory of positive emotions, this study examines whether physical activity is associated with psychological well-being directly and indirectly through these two distinct pathways. Using large-scale national survey data from the 2020 Korean National Survey of Older Persons, the study seeks to clarify how behavioral, interpersonal, and affective processes are jointly associated with well-being among older adults in contemporary Korean society.

## 2. Literature Review

### 2.1. Physical Activity and Psychological Well-Being in Later Life

Physical activity is consistently recognized as an important behavioral factor associated with psychological well-being in later life. Substantial empirical research shows that regular physical activity correlates with greater life satisfaction, improved emotional functioning, and fewer depressive symptoms among older adults (McAuley et al., 2000; Netz et al., 2005; Rejeski & Mihalko, 2001). These associations extend beyond physical health and are also evident in psychological and behavioral domains, including enhanced self-efficacy, better emotional regulation, and a strengthened sense of purpose.

Activity Theory posits that ongoing engagement in meaningful activities is vital for maintaining well-being in later life (Havighurst, 1961). This framework suggests that participation in physical activity may help older adults preserve social roles and identity, both of which are associated with psychological well-being. Further empirical studies in leisure and aging research support this perspective, showing that active involvement in physical and recreational activities is positively linked to subjective well-being (Kuykendall et al., 2015; Pressman et al., 2009). Therefore, both theoretical and empirical evidence supports a positive association between physical activity and psychological well-being.

**H1.**  *Physical activity will be positively associated with psychological well-being.*

### 2.2. Social Contact as a Structural Pathway

Physical activity is increasingly recognized not only for its direct physical benefits but also for its role as a socially embedded behavior that fosters interpersonal interaction and social engagement. When individuals participate in physical activities, they often do so in group or community settings, creating opportunities to connect with others and build social ties. Research has shown that social relationships significantly influence both participation in physical activity and psychological well-being, and that higher levels of social contact are associated with improved mental health, reduced mortality risk, and increased life satisfaction (Berkman & Glass, 2000; Holt-Lunstad et al., 2010; Lindsay-Smith et al., 2019; Park et al., 2024). Social integration theories similarly suggest that individuals deeply embedded in social networks are more likely to feel a sense of belonging and purpose.

Social contact and positive affect represent different but potentially complementary pathways. Social contact reflects the extent to which individuals remain connected through interactions and kinship ties, whereas positive affect reflects the presence of pleasant emotional experiences. Physical activity may support social contact by creating or sustaining opportunities for interaction, and it may also be associated with positive affect through enjoyment, activation, perceived competence, and other emotional responses to activity. Examining these pathways separately avoids treating affective experience as a proxy for social support or relationship quality. Given that physical activity often occurs in contexts that provide opportunities for interpersonal interaction, a positive indirect association between physical activity and psychological well-being through social contact is theoretically plausible.

**H2.**  *Physical activity will show a positive indirect association with psychological well-being through social contact.*

### 2.3. Affective Dimension: Positive Affect as a Mediator

Beyond structural social contact, physical activity may also be associated with psychological well-being through affective mechanisms. Subjective well-being is widely conceptualized as a tripartite construct comprising life satisfaction, positive affect, and negative affect, which are correlated but partially distinct components of overall well-being (Diener et al., 1999, 2018). Whereas life satisfaction reflects a cognitive, evaluative judgment about one’s life circumstances, positive affect reflects the frequency and intensity of pleasant emotional experiences, such as feeling refreshed, joyful, or glad to be alive. Because positive affect and evaluative well-being are theoretically and empirically distinguishable components of subjective well-being rather than the same construct measured twice, positive affect can meaningfully be examined as a mediator of the association between physical activity and the evaluative, life-satisfaction-based psychological well-being construct used in this study.

Physical activity is theorized to elevate positive affect through several mechanisms, including the physiological effects of exercise, the intrinsic enjoyment of engaging in valued activities, and the social context in which much physical activity among older adults takes place. According to the broaden-and-build theory of positive emotions, positive affective states broaden individuals’ momentary thought–action repertoires and help build enduring social and psychological resources over time (Fredrickson, 2001). Group-based or socially embedded physical activity, such as community walking groups or recreational exercise programs, may therefore constitute a context in which physical activity and positive affect reinforce one another. Consistent with this reasoning, physical activity has been repeatedly linked to elevated positive affect and reduced depressive symptoms among older adults (Bragina & Voelcker-Rehage, 2018; McAuley et al., 2000).

In turn, positive affect is theorized to be associated with greater psychological well-being both directly, as a core affective component of subjective well-being, and indirectly, by supporting engagement, motivation, and resilience in daily life (Thoits, 2011). Based on this theoretical perspective and previous empirical findings, a positive indirect association between physical activity and psychological well-being through positive affect is expected among older adults.

**H3.**  *Physical activity will show a positive indirect association with psychological well-being through positive affect.*

### 2.4. Conceptual Framework

**Figure 1:**
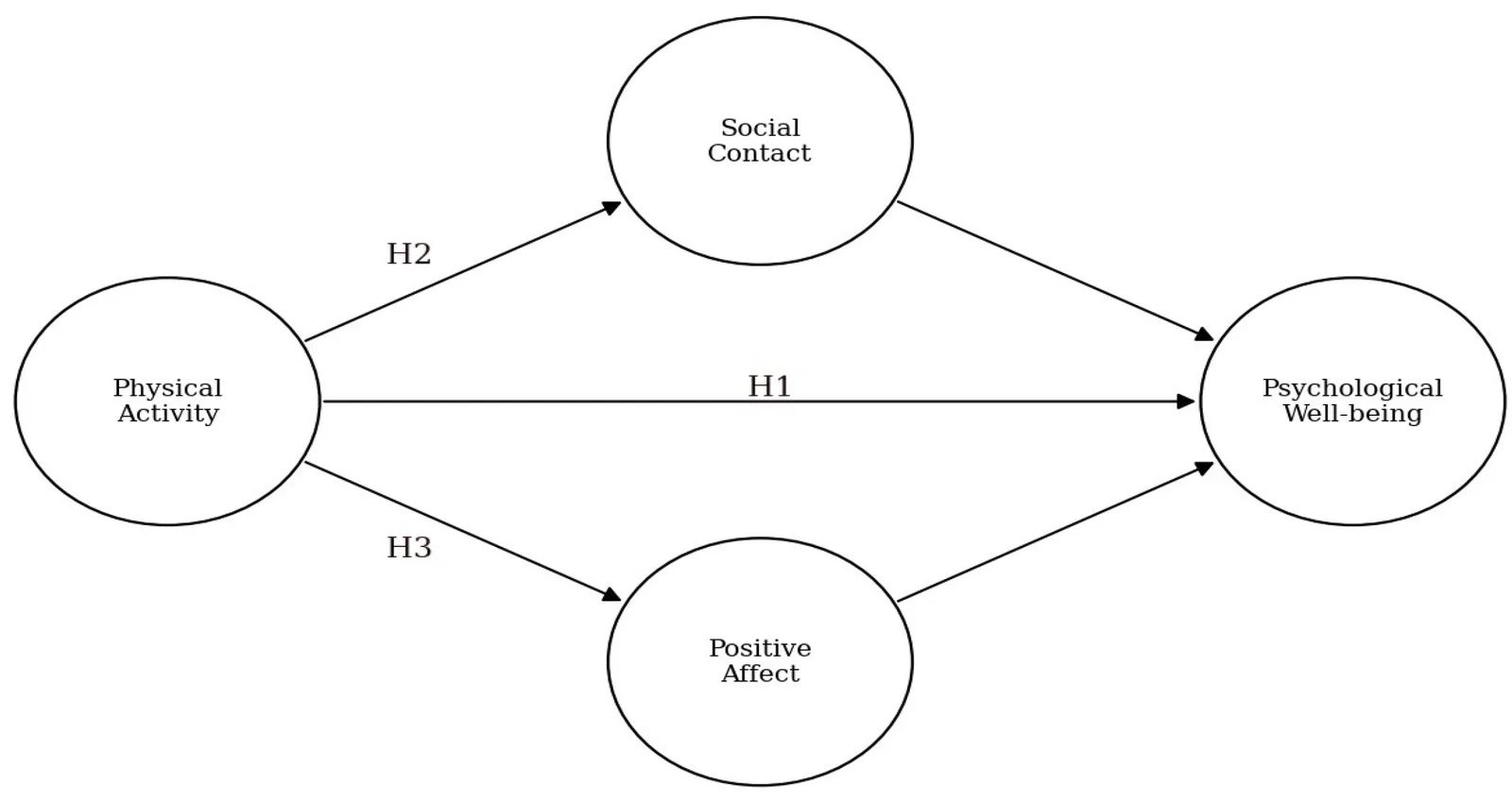
Theoretical Dual-Pathway Mediation Model and Hypothesized Directions.

This study proposes a dual social–affective pathway model based on the hypotheses above. The model examines whether physical activity is associated with psychological well-being directly and indirectly through (1) social contact, representing an interpersonal pathway, and (2) positive affect, representing an affective pathway. The two mediators are expected to be related but conceptually distinct, allowing the model to assess their complementary contributions without assuming that positive affect is itself a measure of social support (Figure 1).

## 3. Methods

### 3.1. Data Source and Participants

This study utilized data from the 2020 Korean National Survey of Older Persons (KNSoP), a nationally representative survey conducted by the Ministry of Health and Welfare and the Korea Institute for Health and Social Affairs. The KNSoP employed a stratified multistage cluster sampling procedure based on administrative districts and housing types to ensure adequate representation of adults aged 65 years and older across all regions of South Korea. Primary sampling units were first selected within strata, followed by household selection and participant recruitment.

The original survey included 10,097 respondents. Descriptive statistics were calculated using the full survey sample (N = 10,097). For correlation analyses and structural equation modeling, cases with missing values for the study variables were excluded using listwise deletion, resulting in an analytical sample of 9551 participants.

Missingness was handled in two steps. Structurally inapplicable responses were first recoded to substantively appropriate values: respondents reporting no regular exercise were coded as 0 for exercise frequency and duration, respondents with no close relatives were coded as 0 for number of close relatives, and respondents with no living siblings or relatives were assigned the lowest-contact category for contact frequency. Item-level nonresponse was then handled by listwise deletion. The resulting case flow was as follows: the original sample comprised 10,097 respondents; after structurally inapplicable responses were recoded, 546 cases with remaining item-level nonresponse were excluded, leaving 9551 complete cases for correlation and SEM analyses (94.6% of the original sample).

The present study involved secondary analysis of publicly available, de-identified survey data. No direct contact with participants occurred during the research process, and no personally identifiable information was accessible to the authors at any stage of the research.

### 3.2. Measures

#### 3.2.1. Physical Activity

Physical activity was assessed using three indicators derived from the KNSoP dataset: participation in regular exercise, exercise frequency, and exercise duration. These indicators reflect behavioral engagement in planned and structured physical activities and are consistent with commonly used measures of physical activity participation in aging research (Penedo & Dahn, 2005; Warburton et al., 2006). Previous studies have suggested that regular participation, frequency, and duration represent key behavioral dimensions of habitual physical activity among older adults and are associated with both physical and psychological health outcomes. Higher scores indicate greater engagement in physical activity. The construct demonstrated satisfactory psychometric properties (CR = 0.92, AVE = 0.80, Cronbach’s α = 0.92). Specifically, respondents were asked whether they engaged in regular exercise. Those who reported regular exercise were then asked how many days per week they exercised and how many minutes they exercised per session. Thus, the three indicators represented regular-exercise participation, weekly exercise frequency, and exercise duration per session.

#### 3.2.2. Social Contact

Social contact was operationalized using two indicators reflecting contact with family and relatives: frequency of contact with siblings and relatives, and the number of close relatives. A third indicator originally considered for this construct, frequency of contact with friends and neighbors, was excluded following confirmatory factor analysis: this item showed a standardized loading of only 0.21 on the intended factor and substantial cross-loading with the psychological well-being indicator assessing satisfaction with friend and community relationships, indicating that it captured relationship satisfaction more than the structural frequency of contact it was intended to measure. Consistent with social network and social integration research, the retained indicators represent the structural dimension of social relationships because they reflect the extent to which individuals remain connected within family and kinship networks (Berkman & Glass, 2000; Umberson & Montez, 2010). Frequency of contact has been widely used as an indicator of social integration and social participation among older adults and is associated with greater social connectedness and psychological adjustment (Levasseur et al., 2010). With two indicators, the construct exhibited reliability values below conventional thresholds (CR = 0.47, AVE = 0.31, standardized Cronbach’s α = 0.47); however, discriminant validity was supported, as the square root of AVE exceeded the construct’s correlations with all other constructs. Given the reduced item count, findings related to social contact should be interpreted with appropriate caution. The survey questions asked, in substance, how frequently respondents were in contact with their siblings or other relatives and how many relatives they considered close. These questions were used to represent the frequency and extent of kin-based social contact.

#### 3.2.3. Positive Affect

Positive affect was measured using three items drawn from the positive-affect subscale of the KNSoP short-form depression screening module, assessing whether respondents had recently felt refreshed, felt joy, and felt glad to be alive. These items directly assess the affective component of subjective well-being—the frequency of pleasant emotional experience—consistent with the tripartite conceptualization of subjective well-being as comprising life satisfaction, positive affect, and negative affect (Diener et al., 1999). Higher scores indicate greater positive affect. Because these items originate from a depression-screening instrument, their wording was not designed specifically for positive-affect research; nonetheless, all three items directly assess the presence of pleasant affective experience, rather than perceived or received social support, which is consistent with their use here as indicators of positive affect. In addition, the three binary items capture only a limited range of pleasant affective experiences and may not fully represent the breadth, frequency, or intensity of positive affect. This reflects a reconceptualization of this construct, in the present version of the manuscript, as a component of subjective well-being rather than as a measure of received or perceived emotional support, addressing a construct-validity concern raised in an earlier review. The construct demonstrated acceptable reliability (CR = 0.73; Cronbach’s α = 0.73), although its AVE (0.47) was slightly below the conventional 0.50 criterion for convergent validity. Representative questions asked whether respondents had recently felt refreshed, experienced joy, and felt glad to be alive; each was answered in a binary yes/no format.

This reconceptualization also addresses a potential concern regarding conceptual circularity between positive affect and psychological well-being—that is, whether the strong association observed between these constructs in the structural model (Section 4.4) simply reflects two measures of the same underlying construct rather than a substantive relational pathway. Three considerations speak against this interpretation. First, the two constructs differ in content: the positive affect items ask about general affective experience (feeling refreshed, joyful, or glad to be alive) using a binary yes/no format, whereas the psychological well-being items ask about domain-specific evaluative judgments (satisfaction with health, economic status, family relationships, social and leisure activities, and life overall) using a five-point satisfaction scale; the two sets of items differ in both content and response format, which limits the extent to which their association can be attributed to shared method variance. Second, the confirmatory factor analysis supported discriminant validity between the two constructs, as reported in Section 4.2: the square root of AVE for positive affect (0.69) and for psychological well-being (0.71) both exceeded their inter-construct correlation (0.49), indicating that each construct captures more variance from its own indicators than it shares with the other. Third, a correlation of this magnitude between positive affect and life-satisfaction-based well-being measures is consistent with the broader subjective well-being literature, in which meta-analytic evidence indicates that positive affect and life satisfaction are moderately, not perfectly, correlated components of a common but multidimensional subjective well-being construct (Busseri, 2018). Taken together, these considerations support treating positive affect and psychological well-being as related but empirically distinguishable constructs, consistent with their theoretical conceptualization in Section 2.3.

#### 3.2.4. Psychological Well-Being

Psychological well-being was assessed using six indicators representing evaluative satisfaction with major life domains and life overall. These indicators correspond closely to the conceptualization of subjective well-being proposed by Diener and colleagues, which emphasizes individuals’ cognitive and affective evaluations of their lives (Diener et al., 2018). Such measures have been widely used in gerontological research and are considered core indicators of psychological well-being among older adults. The construct demonstrated strong reliability and convergent validity (CR = 0.86, AVE = 0.51, Cronbach’s α = 0.86). The six questions asked respondents to rate their satisfaction with major life domains, including health, economic status, relationships with children, relationships with friends and the community, social and leisure activities, and life overall. For example, respondents were asked how satisfied they were with their health and with their life overall, using a five-point satisfaction scale.

### 3.3. Measurement Validity and Reliability

A confirmatory factor analysis (CFA) was conducted to evaluate the adequacy of the measurement model. Model fit was assessed using multiple indices, including the Comparative Fit Index (CFI), Tucker–Lewis Index (TLI), Root Mean Square Error of Approximation (RMSEA), and Standardized Root Mean Square Residual (SRMR). Following commonly accepted criteria, CFI and TLI values above 0.90 and RMSEA and SRMR values below 0.08 were considered indicative of acceptable model fit (Hu & Bentler, 1999). Convergent validity was assessed using standardized factor loadings, composite reliability (CR), and average variance extracted (AVE). Standardized factor loadings greater than 0.50 and CR values greater than 0.70 were considered acceptable indicators of convergent validity (Hair et al., 2010). An AVE value of 0.50 or higher was considered preferable for convergent validity. The social contact construct fell substantially below conventional reliability and convergent-validity thresholds; consequently, all findings involving this construct were interpreted conservatively. Discriminant validity was evaluated using the Fornell–Larcker criterion (Fornell & Larcker, 1981). Internal consistency reliability was also examined using Cronbach’s alpha coefficients. To assess potential common method bias, Harman’s single-factor test was conducted. The first unrotated factor accounted for less than 50% of the total variance, suggesting that a single common factor was unlikely to account for most of the covariance among the measures; however, shared method variance cannot be ruled out by this diagnostic alone.

### 3.4. Data Analysis

Primary analyses were conducted using IBM SPSS Statistics 26.0 and AMOS 26.0. A supplementary covariate-adjusted robustness analysis was conducted in Python 3.12.7 using semopy 2.3.11. First, descriptive statistics and correlation analyses were performed to examine sample characteristics and bivariate associations among study variables. Second, confirmatory factor analysis was conducted to evaluate the measurement model. Third, structural equation modeling (SEM) was used to examine the hypothesized associations among physical activity, social contact, positive affect, and psychological well-being. Because the present study utilized cross-sectional secondary data, the estimated structural paths should be interpreted as statistical associations rather than causal effects. Accordingly, terms such as “association,” “relationship,” and “indirect association” are used throughout the manuscript in place of causal terminology. Finally, indirect associations were examined using a bootstrapping procedure with 5000 resamples. Indirect effects were considered statistically significant when the 95% bias-corrected confidence interval did not include zero. Because the primary analyses focused on latent-variable associations rather than population prevalence estimates, the SEM was estimated at the case level without applying survey weights. Accordingly, the reported coefficients should be interpreted as unweighted associations within the analytic sample rather than as population-weighted parameters.

## 4. Results

### 4.1. Participants

The characteristics of the study participants are presented in Table 1. In terms of gender distribution, women (60.0%) comprised a higher proportion than men (40.0%). The overall age distribution consisted of 3521 individuals (34.9%) aged 65–69, 2492 (24.7%) aged 70–74, 1988 (19.7%) aged 75–79, 1424 (14.1%) aged 80–84, and 672 (6.7%) aged 85 years and older. In terms of residential area, the largest proportion of participants resided in the Gyeongsang region (30.2%), followed by Chungcheong-do (17.2%), Jeolla-do (15.9%), Gyeonggi-do (11.8%), and Seoul (10.4%), indicating a broad regional distribution across Korea.

Regarding marital status, those with a spouse constituted the majority (58.7%), followed by widowed individuals (37.0%), reflecting the demographic characteristics of the older population. In terms of educational attainment, the highest proportion was among elementary school graduates (33.4%), while middle school (23.5%) and high school graduates (26.4%) were relatively evenly distributed. Individuals with a university degree or higher represented a comparatively smaller proportion.

Regarding subjective health status, most participants reported their health as ‘Good’ (45.4%) or ‘Fair’ (31.5%), while 16.7% reported ‘Poor’ health. Homeownership was the predominant housing type (79.7%). Regarding economic activity, the largest proportion reported having worked in the past but not currently (48.6%). The distribution of chronic diseases indicated that many older adults experienced multiple health conditions, with 29.3% reporting one disease, 27.4% reporting two diseases, and 26.6% reporting three or more diseases.

### 4.2. Measurement Model

As shown in Table 2, the measurement model demonstrated excellent overall fit following the removal of a poorly performing social contact indicator (see Section 3.2.2). Standardized factor loadings for physical activity, positive affect, and psychological well-being ranged from 0.56 to 0.98 and were all statistically significant. Composite reliability (CR) values ranged from 0.73 to 0.92, while average variance extracted (AVE) values ranged from 0.47 to 0.80 for these three constructs.

With only two remaining indicators, the social contact construct exhibited CR (0.47) and AVE (0.31) values below conventional thresholds, and its standardized loadings (0.60 and 0.51) were more modest than those of the other constructs. Discriminant validity was nonetheless supported: the square root of AVE for social contact (0.56) exceeded its correlations with all other constructs. Given the reduced item count and modest convergent validity, findings involving social contact should be interpreted with caution.

The measurement model indicated that the four constructs were conceptually and empirically distinguishable. However, because the social contact construct showed relatively modest AVE and reliability values, results involving this construct should be interpreted cautiously.

#### Discriminant Validity

Discriminant validity was evaluated using the Fornell–Larcker criterion, which compares the square root of the average variance extracted (AVE) for each construct against its correlations with all other constructs (Fornell & Larcker, 1981). A construct demonstrates discriminant validity when the square root of its AVE exceeds its correlations with every other construct in the model, indicating that it shares more variance with its own indicators than with those of other constructs. As shown in Table 3, the diagonal values (the square root of AVE for each construct) exceeded the corresponding inter-construct correlations in every case. This pattern held even for social contact, the construct with the lowest AVE (0.31): its diagonal value (0.56) still exceeded its correlations with physical activity (0.14), positive affect (0.16), and psychological well-being (0.29). Discriminant validity was therefore supported for all four constructs, indicating that they are empirically distinguishable despite the modest convergent validity of the social contact construct.

### 4.3. Correlation

Descriptive statistics and Pearson correlation coefficients are presented in Table 4. Overall, the correlations among the study variables were statistically significant and generally in the expected directions. Physical activity was significantly associated with both social contact and positive affect, and both constructs were significantly associated with psychological well-being. None of the correlation coefficients were sufficiently large to indicate potential multicollinearity concerns. Composite scores for each variable were computed as the mean of standardized (z-score) item indicators, with item direction aligned so that higher scores indicate more physical activity, more social contact, more positive affect, and greater psychological well-being. Because each composite is the arithmetic mean of standardized items, its M is, by construction, equal to 0.00 in standardized units; the corresponding SD values are less than 1.00 (ranging from 0.763 to 0.929), reflecting the degree of intercorrelation among the component items for each construct.

These findings provide preliminary support for the proposed relationships among the study variables and justify the subsequent structural equation modeling analysis.

### 4.4. Structural Model

The structural model was estimated to examine the hypothesized relationships among physical activity, social contact, positive affect, and psychological well-being, using the revised two-indicator social contact construct described in Section 3.2.2. As shown in Table 5, the model demonstrated a good fit to the data (χ^2^ = 1321.777, df = 72, CFI = 0.978, TLI = 0.972, RMSEA = 0.043, 90% CI [0.041, 0.045], N = 9551).

Physical activity was positively associated with social contact (β = 0.147, *p* < 0.001) and positive affect (β = 0.155, *p* < 0.001). In turn, social contact (β = 0.237, *p* < 0.001) and positive affect (β = 0.481, *p* < 0.001) were positively associated with psychological well-being. The direct association between physical activity and psychological well-being was small and not statistically significant (β = 0.019, *p* = 0.077). The model explained 2.2% of the variance in social contact, 2.4% in positive affect, and 29.7% in psychological well-being. Given the limited reliability and convergent validity of the two-indicator social-contact construct, the corresponding structural association should be interpreted more cautiously than the positive-affect pathway.

Taken together, the findings indicate that physical activity was related to psychological well-being primarily through the combined indirect association represented by social contact and positive affect rather than through a statistically significant direct path. Hypothesis 1, which proposed a positive direct association between physical activity and psychological well-being, was therefore not supported.

### 4.5. Mediation Effects

Indirect associations were evaluated using standardized direct, indirect, and total effects from the structural equation model and bias-corrected bootstrapping with 5000 resamples. As shown in Table 6, the standardized total indirect effect of physical activity on psychological well-being through the two mediators combined was statistically significant (β = 0.109, bias-corrected 95% CI [0.094, 0.125], *p* < 0.001).

The product-of-coefficients estimate for the social-contact pathway was approximately 0.035 (0.147 × 0.237), and the corresponding estimate for the positive-affect pathway was approximately 0.075 (0.155 × 0.481). Thus, the positive-affect component was numerically about twice as large as the social-contact component. However, the AMOS output provided a bootstrap confidence interval for the total indirect effect, not separate confidence intervals for the two specific indirect effects or a bootstrap contrast between them. Accordingly, the statistical significance of each specific indirect effect and of the difference between the two components should not be inferred from the point estimates alone.

The direct effect of physical activity on psychological well-being was not statistically significant (β = 0.019, bias-corrected 95% CI [−0.002, 0.041], *p* = 0.076), whereas the total effect was significant (β = 0.128, bias-corrected 95% CI [0.107, 0.150], *p* < 0.001). This pattern is consistent with an indirect-only association at the level of the combined indirect effect in the specified cross-sectional model.

**Figure 2:**
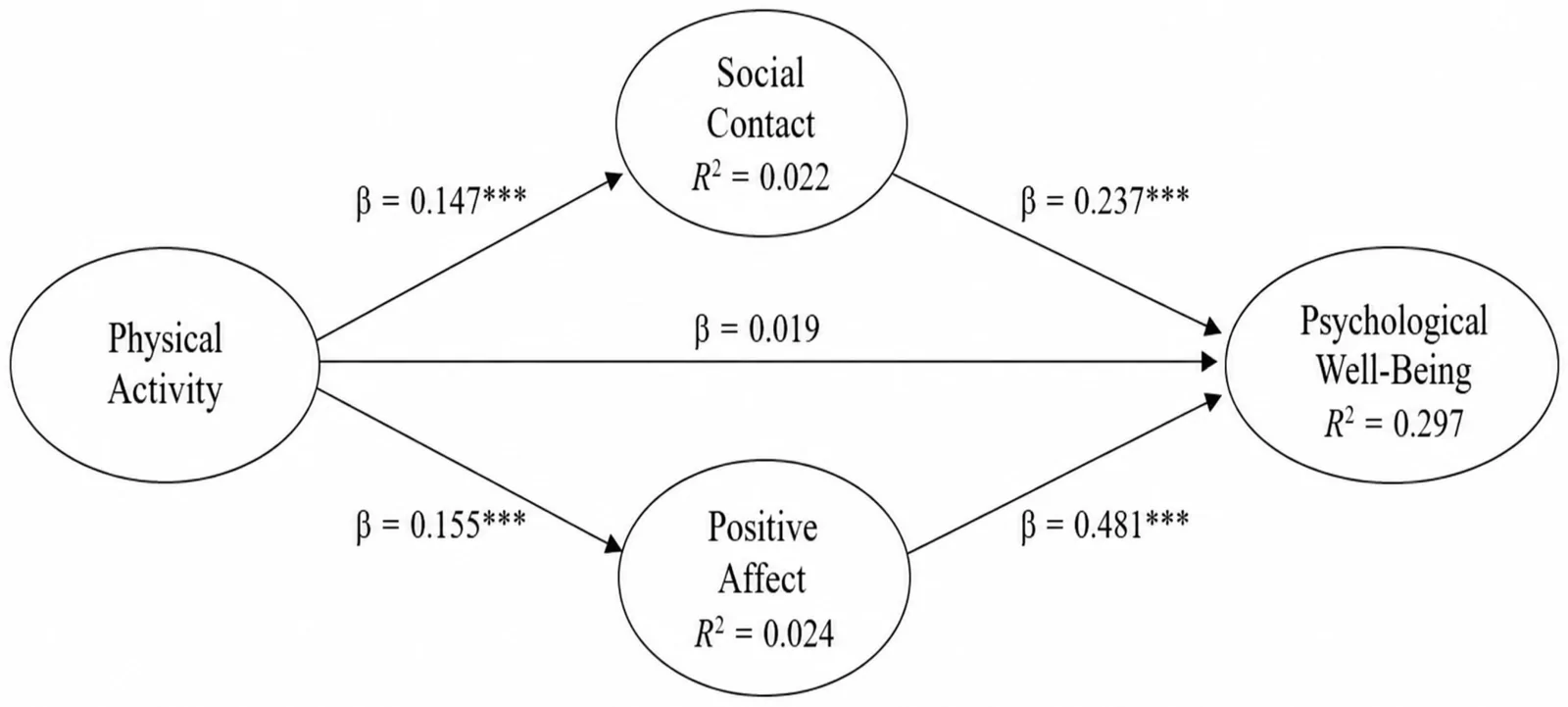
Final Structural Model with Standardized Path Coefficients and Explained Variance (R^2^). *** *p* < 0.001.

The component paths underlying Hypotheses 2 and 3 were statistically significant and in the expected positive direction. Nevertheless, because separate bootstrap confidence intervals were not available for the two specific indirect effects, the findings provide directional support for the proposed social-contact and positive-affect pathways while formally establishing significance only for their combined total indirect effect. These associations should not be interpreted as demonstrated causal mediation (Figure 2).

### 4.6. Robustness Check: Covariate-Adjusted Model

To assess robustness to demographic and health-related confounding, a supplementary model included age, gender, marital status, education, subjective health status, number of chronic diseases, economic activity status, and homeownership as predictors of social contact, positive affect, and psychological well-being. The supplementary analysis used the same indicators, analytic sample (N = 9551), and focal structural specification as the primary model, but was estimated in Python 3.12.7 using semopy 2.3.11. Both models used maximum-likelihood-based estimation; however, because software-specific implementation and scaling procedures may differ, the resulting coefficients and fit indices were not treated as directly interchangeable. The supplementary analysis was therefore used only to examine whether the direction and significance pattern of the focal paths remained similar after covariate adjustment.

As shown in Table 7, the four paths that were significant in the primary model remained significant and in the same direction after covariate adjustment, although their standardized magnitudes were attenuated. The direct path from physical activity to psychological well-being remained non-significant. This consistency supports the robustness of the overall directional pattern, but it should not be interpreted as confirming identical parameter estimates across AMOS and semopy or as overcoming the measurement limitations of the social-contact construct. The covariate-adjusted model showed acceptable fit (χ^2^ = 5906.354, df = 196, CFI = 0.926, TLI = 0.910, RMSEA = 0.055).

## 5. Discussion

The present study examined associations among physical activity, social contact, positive affect, and evaluative psychological well-being among older adults in Korea. The combined indirect association through social contact and positive affect was significant, whereas the direct association between physical activity and psychological well-being was not. Product-of-coefficients estimates were approximately 0.035 for the social-contact component and 0.075 for the positive-affect component. Because separate bootstrap confidence intervals and a formal contrast were unavailable, these component estimates are descriptive and do not establish that either specific indirect pathway was independently significant or statistically stronger.

The nonsignificant direct path suggests that physical activity and psychological well-being may be associated within broader interpersonal and affective contexts. This interpretation is consistent with evidence linking physical activity to later-life well-being (Bragina & Voelcker-Rehage, 2018), while longitudinal evidence also suggests that psychological well-being can predict later physical activity (Kim et al., 2017). The present cross-sectional model therefore does not establish temporal ordering or a unidirectional causal process; reverse or reciprocal relationships remain plausible.

Social contact represented the interpersonal pathway: physical activity was positively associated with social contact, which was positively associated with psychological well-being. This pattern is consistent with research linking social interaction and participation with life satisfaction and quality of life among older adults, including Korean populations (Choi et al., 2020; Park & Kang, 2023). However, the social-contact construct retained only two kin-based indicators and showed below-threshold reliability and convergent validity. This branch should therefore be interpreted conservatively and should not be generalized to broader community or friendship networks.

Positive affect represented the affective pathway. Physical activity was positively associated with positive affect, which in turn showed a comparatively strong association with evaluative psychological well-being. Pleasant emotional experience and life satisfaction are related but distinguishable components of subjective well-being, although their conceptual proximity may partly contribute to the observed association. The results therefore support an affective interpretation but do not demonstrate emotional support or relationship quality, which were not directly measured.

Together, the interpersonal and affective patterns provide the study’s main theoretical contribution by distinguishing relational connectedness from positive emotional experience rather than treating psychosocial correlates of physical activity as a single mechanism. In the Korean context, this distinction suggests that physical activity may be associated with later-life well-being across more than one psychosocial dimension, while the relative strength and independence of these pathways require further study.

The findings may have practical relevance, but the cross-sectional design does not establish the effectiveness of any specific intervention or program feature. Rather, the observed associations highlight the potential relevance of considering interpersonal and affective contexts when examining physical activity among older adults. Group exercise, walking programs, and community recreation may provide opportunities for social interaction and enjoyable participation; however, longitudinal and intervention studies are needed to determine whether such program features lead to improvements in psychological well-being.

### Limitations and Future Research

Several limitations warrant consideration. The cross-sectional design prevents causal or temporal conclusions, so the indirect effects represent statistical associations rather than demonstrated mediation mechanisms. All focal variables were self-reported, and shared method variance or response-related bias cannot be excluded. Social contact was also measured with only two retained indicators and showed low reliability and convergent validity; findings involving this construct should therefore be interpreted conservatively.

The scope of the model also requires caution. Physical activity explained only 2.2% of the variance in social contact and 2.4% in positive affect, indicating that most variance in these mediators lies outside the model. AMOS also provided a bootstrap confidence interval only for the combined total indirect effect, not for each specific indirect component. In addition, the primary SEM used unweighted case-level data rather than reproducing the survey’s complex population-weighting scheme. Thus, the estimates characterize associations within the analytic sample and should not be interpreted as population-weighted parameters.

Future research should use longitudinal or intervention designs to clarify temporal ordering and reciprocal relationships, apply more comprehensive measures of social contact and purpose-built measures of positive affect, and estimate specific indirect effects with separate bootstrap confidence intervals. Replication with survey-weighted analyses and in other cultural settings would further strengthen generalizability.

## 6. Conclusions

Using data from the 2020 Korean National Survey of Older Persons, this study found that physical activity was positively associated with both social contact and positive affect, and that both were positively associated with evaluative psychological well-being. The direct association between physical activity and psychological well-being was not statistically significant, whereas the combined total indirect association through the two pathways was significant.

These findings are consistent with a differentiated social–affective perspective in which physical activity is considered within a broader interpersonal and emotional context rather than as an isolated behavior. However, the social-contact branch should be interpreted cautiously because it was based on a two-indicator construct with below-threshold reliability and convergent validity. The findings therefore suggest the potential relevance of interpersonal and affective contexts of physical activity among older adults, but they do not establish the effectiveness of specific programs or interventions. Longitudinal and intervention studies are required before specific program recommendations can be made.

## Figures and Tables

**Table 1:** Descriptive characteristics of the study participants.

Variable	Category	N	%
Gender	Male	4035	40.0
	Female	6062	60.0
Age (years)	65–69	3521	34.9
	70–74	2492	24.7
	75–79	1988	19.7
	80–84	1424	14.1
	≥85	672	6.7
Marital status	Single	43	0.4
	Married	5931	58.7
	Widowed	3737	37.0
	Divorced	330	3.3
	Separated	56	0.6
Residential area	Seoul	1049	10.4
	Incheon	563	5.6
	Gyeonggi	1190	11.8
	Gyeongsang	3045	30.2
	Jeolla	1607	15.9
	Chungcheong	1733	17.2
	Gangwon	511	5.1
	Jeju	399	4.0
Education level	No Formal Education	1171	11.6
	Elementary School	3377	33.4
	Middle School	2369	23.5
	High School	2668	26.4
	Junior College	203	2.0
	University or Higher	309	3.1
Subjective health status	Very Good	433	4.4
	Good	4507	45.4
	Fair	3120	31.5
	Poor	1659	16.7
	Very Poor	201	2.0
Spouse’s health status	Very Good	347	5.9
	Good	3128	52.7
	Fair	1650	27.8
	Poor	683	11.5
	Very Poor	122	2.1
Housing type	Owned	8044	79.7
	Jeonse (Lease Deposit)	818	8.1
	Monthly Rent	752	7.4
	Monthly Rent (Low-cost)	82	0.8
	Rent-Free	401	4.0
Economic activity	Currently Employed	3785	37.5
	Previously Employed	4908	48.6
	Not Employed	1404	13.9
Number of chronic diseases	0	1686	16.7
	1	2956	29.3
	2	2766	27.4
	≥3	2689	26.6

Note: Frequencies are reported based on available responses for each variable. The full survey sample included 10,097 respondents. Correlation and structural equation modeling analyses were conducted using complete cases (N = 9551).

**Table 2:** Results of confirmatory factor analysis of measurement model.

Construct	Item	Loading	CR	AVE	Cronbach’s α
Physical activity	PA1	0.98	0.92	0.80	0.92
	PA2	0.90			
	PA3	0.80			
Social contact	SC1	0.60	0.47	0.31	0.47
	SC2	0.51			
Positive affect	PAf1	0.66	0.73	0.47	0.73
	PAf2	0.68			
	PAf3	0.72			
Psychological well-being	WB1	0.68	0.86	0.51	0.86
	WB2	0.71			
	WB3	0.56			
	WB4	0.74			
	WB5	0.71			
	WB6	0.85			
Model fit: χ^2^ = 628.391, df = 66, CFI = 0.990, TLI = 0.986, RMSEA = 0.030, GFI = 0.991 (N = 9551)					

**Table 3:** Discriminant validity (Fornell–Larcker criterion).

Construct	1	2	3	4
1. Physical activity	**0.90**			
2. Social contact	0.14	**0.56**		
3. Positive affect	0.15	0.16	**0.69**	
4. Psychological well-being	0.13	0.29	0.49	**0.71**

Note: Diagonal values in bold represent the square root of the average variance extracted (AVE) for each construct; off-diagonal values represent correlations among the latent constructs estimated in the confirmatory factor analysis. Discriminant validity is supported when the diagonal value in each row and column exceeds the corresponding off-diagonal values.

**Table 4:** Correlations among study variables.

Variable	M	SD	1	2	3	4
1. Physical activity	0.00	0.929	1			
2. Social contact	0.00	0.809	0.115 ***	1		
3. Positive affect	0.00	0.805	0.139 ***	0.095 ***	1	
4. Psychological well-being	0.00	0.763	0.125 ***	0.194 ***	0.402 ***	1

*** *p* < 0.001.

**Table 5:** Structural paths.

Path	B	SE	t-Value	*p*-Value	β	Result
Physical activity → Social contact	0.004	<0.001	9.382	<0.001	0.147	Supported
Physical activity → Positive affect	0.002	<0.001	12.563	<0.001	0.155	Supported
Social contact → Psychological well-being	0.197	0.016	12.208	<0.001	0.237	Supported
Positive affect → Psychological well-being	0.879	0.027	32.775	<0.001	0.481	Supported
Physical activity → Psychological well-being	<0.001	<0.001	1.768	0.077	0.019	Not Supported
Model fit: χ^2^ = 1321.777, df = 72, CFI = 0.978, TLI = 0.972, RMSEA = 0.043 (N = 9551)						

Note: B = unstandardized regression coefficient; SE = standard error; β = standardized regression coefficient. Because the raw indicators differ substantially in scale, some unstandardized coefficients and standard errors round to less than 0.001 at three decimal places even though they are non-zero. Standardized coefficients are therefore emphasized when comparing path magnitudes. The endogenous-variable R^2^ values were 0.022 for social contact, 0.024 for positive affect, and 0.297 for psychological well-being.

**Table 6:** Direct, Indirect, and Total Effects of Physical Activity on Psychological Well-being.

Effect	Standardized Effect	Bias-Corrected 95% CI	*p*
Direct effect (PA → PWB)	0.019	[−0.002, 0.041]	0.076
Specific indirect component via social contact	0.035	Not provided	
Specific indirect component via positive affect	0.075	Not provided	
Total indirect effect	0.109	[0.094, 0.125]	<0.001
Total effect	0.128	[0.107, 0.150]	<0.001

Note: Specific indirect components were calculated as products of standardized path coefficients. AMOS provided a bootstrap confidence interval for the total indirect effect but not for the two specific indirect components.

**Table 7:** Covariate-Adjusted Structural Paths (Sensitivity Analysis).

Path	Primary Model β	Covariate-Adjusted β	*p*
Physical activity → Social contact	0.147	0.068	<0.001
Physical activity → Positive affect	0.155	0.103	<0.001
Social contact → psychological well-being	0.237	0.136	<0.001
Positive affect → Psychological well-being	0.481	0.310	<0.001
Physical activity → Psychological well-being (direct)	0.019	−0.002	0.809

Note: Covariates: age, gender, marital status, education, subjective health status, number of chronic diseases, economic activity status, and homeownership. Covariate-adjusted estimates were obtained from a supplementary model estimated in Python (semopy) using the same analytic sample and indicators as the primary AMOS-based model; they are reported as a robustness check rather than as the primary results of this study.

## Data Availability

The data used in this study are publicly available from the Korean National Survey of Older Persons through the Korea Institute for Health and Social Affairs (KIHASA).
